# kESVR: An Ensemble Model for Drug Response Prediction in Precision Medicine Using Cancer Cell Lines Gene Expression

**DOI:** 10.3390/genes12060844

**Published:** 2021-05-30

**Authors:** Abhishek Majumdar, Yueze Liu, Yaoqin Lu, Shaofeng Wu, Lijun Cheng

**Affiliations:** 1 Department of Biomedical Informatics, College of Medicine, The Ohio State University, Columbus, OH 43210, USA; abhishek.majumdar@osumc.edu (A.M.); wu.2946@buckeyemail.osu.edu (S.W.); 2 The Grainger College of Engineering, The University of Illinois Urbana-Champaign, Urbana and Champaign, Champaign, IL 61801, USA; yuezel2@illinois.edu; 3 Department of Occupational and Environmental Health, School of Public Health, XinJiang Medical University, Urumqi 830011, China; lyq_superior@163.com

**Keywords:** cancer cell-lines, gene expression, drug response prediction

## Abstract

Background: Cancer cell lines are frequently used in research as in-vitro tumor models. Genomic data and large-scale drug screening have accelerated the right drug selection for cancer patients. Accuracy in drug response prediction is crucial for success. Due to data-type diversity and big data volume, few methods can integrative and efficiently find the principal low-dimensional manifold of the high-dimensional cancer multi-omics data to predict drug response in precision medicine. Method: A novelty k-means Ensemble Support Vector Regression (kESVR) is developed to predict each drug response values for single patient based on cell-line gene expression data. The kESVR is a blend of supervised and unsupervised learning methods and is entirely data driven. It utilizes embedded clustering (Principal Component Analysis and k-means clustering) and local regression (Support Vector Regression) to predict drug response and obtain the global pattern while overcoming missing data and outliers’ noise. Results: We compared the efficiency and accuracy of kESVR to 4 standard machine learning regression models: (1) simple linear regression, (2) support vector regression (3) random forest (quantile regression forest) and (4) back propagation neural network. Our results, which based on drug response across 610 cancer cells from Cancer Cell Line Encyclopedia (CCLE) and Cancer Therapeutics Response Portal (CTRP v2), proved to have the highest accuracy (smallest mean squared error (MSE) measure). We next compared kESVR with existing 17 drug response prediction models based a varied range of methods such as regression, Bayesian inference, matrix factorization and deep learning. After ranking the 18 models based on their accuracy of prediction, kESVR ranks first (best performing) in majority (74%) of the time. As for the remaining (26%) cases, kESVR still ranked in the top five performing models. Conclusion: In this paper we introduce a novel model (kESVR) for drug response prediction using high dimensional cell-line gene expression data. This model outperforms current existing prediction models in terms of prediction accuracy and speed and overcomes overfitting. This can be used in future to develop a robust drug response prediction system for cancer patients using the cancer cell-lines guidance and multi-omics data.

## 1. Introduction

Precision medicine aims to provide individually tailored cancer treatment by considering an individual’s genetic makeup, genomic makeup and clinical information. Emerging Next Generation Sequencing (NGS) techniques and large-scale cancer screening data helps in achieving this goal [1,2]. Databases such as the Cancer Cell Line Encyclopedia (CCLE) [2] provides public access to genomic data over 1000 cancer cell lines by RNA sequencing (RNA-seq; 1019 cell lines), whole-exome sequencing (326 cell lines), whole-genome sequencing (329 cell lines), and reverse-phase protein array (RPPA; 899 cell lines). The Cancer Therapeutics Response Portal (CTRP; http://portals.broadinstitute.org/ctrp/, accessed on 1 June 2019) [3,4] quantitatively measured the sensitivity of 481 small-molecule probes and drugs. An important step of this process is to use cancer cell line models to simulate mixed tissue and predict his/her drug response [5].

Accuracy in drug response prediction is of utmost importance in this regard. Over the years various models have been developed for this purpose [6,7,8,9]. Contemporary models are based on a varied range of techniques such as regression methods, Bayesian inference methods, matrix factorization methods and deep learning methods. Some of these methods use only gene expression data while some use combination of other omics data such as mutation, copy number variation, methylation and so on for response prediction. Prediction models by gene expression profiles show the best performance in all kinds of omics analysis [10]. The detailed analysis and comparison of different methods can be found in a paper by Chen and Zhang [11].

These models, however, only focus on a single model [12] trained over large datasets. Without recognition of the weekly predictive local embedding data contribution, will cause them to make incorrect decisions in facing to outliers and errors. Outliers’ and errors’ in turn, causes these models to be incapable of capturing the dataset’s true variance, thus distorting model complexity [13]. To deal with high-dimensional genomics data, a promising strategy is to find an effective low-dimensional subspace of the original data and cluster samples in the reduced subspace [14], and then do a localize regression.

However, it is hard to identify the homology features which one contribution to drug response prediction for increasingly heterogeneous datasets comprised of multi-omics data collected from overlapping latent low-dimensional subpopulations. Principal Component Analysis (PCA) can generate statistically uncorrelated principal components (PC) while retaining as much as possible of the variation present in the original data set. PCA has been used previously to delineate homogenous regions by PCs regression and applying in all kinds of fields such as temperature [15], hydrology [16], risk [17], and animal health [18], but have not applied in drug response prediction.

Machine learning nonlinear regression Support Vector Regression (SVR) was first introduced by Vapnik [19] and has been a highly effective and suitable method for regression [20]; the problem of regression is to find a function surface in high dimension that approximates mapping from an input domain (low dimension) to real values based on a training sample. However, most existing SVR learning algorithms are limited to the parameters selection and slow learning for high-throughput features and large samples [21,22].

To address these challenges, a novelty k-means Ensemble Support Vector Regression (kESVR) is developed to predict each drug response values for single patient based on cell-line gene expression data. kESVR’s origin stems from previous work interval SVR [22], where we separated a global nonlinear SVR predictor into interval subspaces and ran a SVR in each interval subspace. However, kESVR is different to the interval SVR in that it constructs a local SVR regression in each principal component embedding subspaces, where the K-means algorithm clusters these homogenous regions and then predict associated drug response. The prediction process function by repeatedly running the local SVR learning algorithm on various distributions’ clustering over the whole training data, then comparing the regression value produced by these local SVR learners to obtain a single regression value with the best performance in accuracy and output. The last step used a boosting strategy as literature [23] mentioned to obtain the high accuracy of any local SVR learning algorithm.

The kESVR is a blend of supervised and unsupervised learning methods while being an entirely data driven model. It utilizes embedded clustering (PCA and k-means clustering) and local regression (Support Vector Regression) to predict drug response and obtain the global optimal value with the smallest mean squared error (MSE) while overcoming missing data and outliers’ noise. In contrast to classical 17 machine learning models [11] that estimate a single, complex model (or only a few complex models), our results show that kESVR model with PCA-compressed features make the training and validation more efficient in both model accuracy and computational costs, outperforming other machine learning methods in generalization performance. 

## 2. Materials and Methods

### 2.1. Materials

Molecular mRNA data from database Cancer Cell Line Encyclopedia (CCLE; https://portals.broadinstitute.org/ccle, accessed on 1 June 2019) [2], which include 610 gene expression profiles with 20,531 genes [24,25,26] which were tested by an Affymetrix HU133 PLUS2.0 array. The 610 cancer cell lines provide 481 drug sensitivity responses in Cancer Therapeutics Response Portal (CTRP *v*2, 2015; http://portals.broadinstitute.org/ctrp, accessed on 1 June 2019) [3,4] which comprises of 70 U.S. Food and Drug Administration (FDA) approved drugs, 100 experimental compounds, and 311 small molecule probes, about half of which has no identified protein targets. AUC (Area Under drug response Curve) was used to assess the extent of exposure of a drug.

### 2.2. Method

#### 2.2.1. Overview

**Figure 1:**
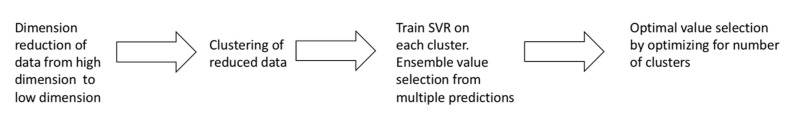
Overview of kESVR model.

k-means Ensemble Support Vector Regression (kESVR) model consists of 4 distinct steps. In this section we provide a brief overview of the different steps comprising our model. Figure 1 summarizes the 4 steps of our model.

1. Dimensional reduction: In the first step, we convert high dimensional gene expression data to low dimensional data for better handling and visualization in the subsequent steps.
2. Embedded Clustering: In the second step, we split the lower dimensional data into distinct clusters based on the their labeled/given drug response value. This is the done so that data points (cell-lines) that have similar or close drug response values are grouped together.
3. Local regression and ensemble value selection: In the third step, we train different instances of a machine learning (ML) model on each of the different clusters of data points obtained in the previous step. If there are k clusters, we train k instances of the ML model. For a given/new input, we now have k candidate ML prediction outputs to select from. We use a score-based approach to select the best output. We base our scoring system on the similarity of gene expression profiles between the input and the training data to get the best prediction result.
4. Optimal drug response value prediction: In the final step, we optimize the number of clusters k to get our model kESVR that gives the best performance (minimum Mean Square Error).

We define the problem of drug response prediction using cell-lines gene expression as follows: Given gene expression data $GE_{g\times n}$ (n: number of cell lines, g: number of genes) and drug response data $R_{d\times n}$ (d: number of drugs, n: number of cell lines), create a prediction model that will accurately predict the response of each of those d drugs for both, the known n cell-lines and unknown cell-line data. 

#### 2.2.2. Generalized Description

##### Dimensional Reduction

Let $CL=\left\{CL_{1},\;CL_{2},\ldots ,\;CL_{N}\right\}$ represent N cancer cell-lines and $X=\left\{X_{1},\;X_{2},\ldots ,\;X_{N}\right\}$ denote the gene expression data of CL. Let $Y=\left\{Y_{1},\;Y_{2},\;\ldots ,\;Y_{N}\right\}$ represent the set of drug response AUC values of CL for a drug D. So, $X_{i}$ is the gene expression data of cell-line $CL_{i}$ and $Y_{i}$ is the drug response of $CL_{i}$. Each $X_{i}$ represents the expression of G genes in the whole genome for $CL_{i}$

(1) $$X_{i}=\left(X_{i}^{1},\;X_{i}^{2},\;\ldots ,\;X_{i}^{G}\right)$$

We use PCA [27] to perform dimensional reduction of X. Let $\left\{\phi_{p}\left(X\right)\right\}$ denote the *p*th Principal Component of X. We select the value of *p* in such a manner that results in minimum loss of information (variation of the data) and use that pth principal component in our subsequent steps. The selection of the principal component can be written as a minimization problem:

$$\underset{p}{\min}\left(var\left[X\right]-var\left[\phi_{p}\left(X\right)\right]\right)$$

Using the pth principal component we create the reduced data,

(2) $$Z=\left\{Z_{1},\;Z_{2},\ldots ,\;Z_{N}\right\}\;\mathrm{where}\;Z_{i}=\left(Y_{i},\phi_{p}\left(X_{i}\right)\right)\;$$

**Figure 2:**
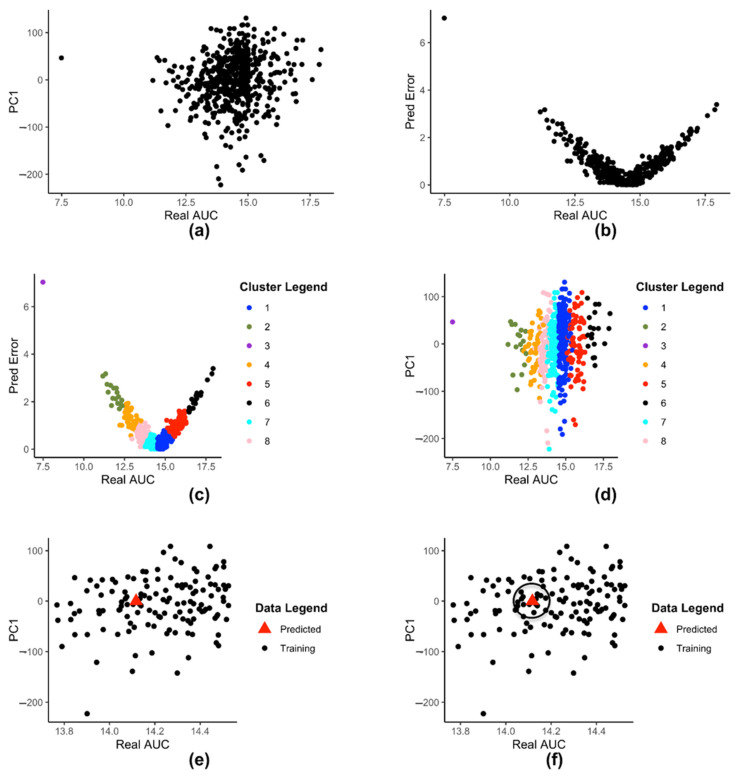
(**a**) 2-D plot of reduced dataset Z (**b**) 2-D representation of prediction error dataset Ψ (**c**) Clustering of Ψ dataset (**d**) 2-D plot of clustered Z (**e**) 2-D plot of data-points in Z with the newly predicted value (**f**) 2-D plot of a predicted data point $\left({\bar{Y}}_{jk},\phi_{p}\left(X_{j}\right)\right)$ and its neighbors.

Figure 2a shows a 2-D plot of reduced dataset Z where the X-axis is the drug AUC value and the Y-axis is the pth principal component value (in this figure p=1). Each point represents a cell-line. All subsequent figures will use p=1.

##### Embedded Clustering

We create a set of labeled data Q using gene expression data X and drug response data Y:

(3) $$Q=\left\{Q_{1},\;Q_{2},\ldots ,\;Q_{N}\right\}\;\mathrm{where}\;Q_{i}=\left\{\left({\hat{X}}_{i},Y_{i}\right)\right\},{\hat{X}}_{i}\subset X_{i}\;\mathrm{and}\;\left|{\hat{X}}_{i}\right|<\left|X_{i}\right|\;$$

Instead of the full set of genes (G) in the whole genome, we use a subset of target genes to create our dataset Q. The elements of Q will be used subsequently in training of support vector regressions (SVR) [19]. We train SVR S, on 75% (random selection) of the labeled data Q. We then use the trained SVR S, to predict the drug response of all the N cell-line gene expressions and calculate the corresponding predicted error values. From this we create the 2-tupled dataset:

(4) $$\Psi =\left\{\left(Y_{1},e_{1}\right),\left(Y_{2},e_{2}\right),\ldots ,\left(Y_{N},e_{N}\right)\right\},$$

where $e_{i}$ denotes the prediction-error obtained from S for input gene expression ${\hat{X}}_{i}$. The tuple $\left(Y_{i},e_{i}\right)$, $Z_{i}$ and $Q_{i}$ are all different representations of the same cell-line $CL_{i}$. We partition the dataset Z into K clusters $G_{1}\ldots G_{K}$ by applying K-means clustering [28] on Ψ.

(5) $$G_{k}=\left\{Z_{k}\right\}\;s.t.\;Z_{k}\in Z\;\mathrm{and}\;\overset{K}{\underset{k=1}{\cup }}G_{k}=Z\;$$

Figure 2b shows the plot of Ψ with drug AUC as X-axis and prediction error as Y-axis. Each point represents a cell-lines. Figure 2c shows Ψ dataset clustered into K=8 clusters after applying K-means clustering. Each cluster is represented by a different color. Figure 2d shows the set of clusters G on a 2-D plane. Each color signifies a different cluster. Figure 2d uses the same color coding as Figure 2c, to show that each color in both figures represent the same cluster of cell-lines.

##### Local Regression and Ensemble Global Value Selection

Let the data point $Z_{j}\in Z$ belong to the cluster $G_{k}$. For each cluster $G_{k}$ we model a SVR $S_{k}$:

$$\;\;\;\;\;\;\;\underset{w_{k},\alpha_{k},\delta_{k}}{\min}\frac{1}{2}{\left\|w_{k}\right\|}^{2}+\alpha_{k}\overset{m}{\underset{j=1}{\sum }}\delta_{jk}$$

$$s.t.\;Y_{j}\left[w_{k}{}^{T}\phi_{p}\left(X_{j}\right)+\beta_{k}\right]\;\geq 1-\delta_{jk},{\;\delta }_{jk}\;\geq 0,j=1\ldots m$$

where $w_{k}$ and $\beta_{k}$ represent the classification hyperplane, $\delta_{k}$ represents the slack variable and $\alpha_{k}$ represents the hyper parameter used for $S_{k}$.

Let ${\bar{Y}}_{jk}$ represent the output predicted by $S_{k}$. So, we have

$${\bar{Y}}_{jk}=\left[w_{k}{}^{T}\phi_{p}\left(X_{j}\right)+\beta_{k}\right].$$

Figure 2e shows a 2-D rendering of the newly predicted value ${\bar{Y}}_{jk}$ produced by input $X_{j}$ when plotted as a point $\left({\bar{Y}}_{jk},\phi_{p}\left(X_{j}\right)\right)$ along with the rest of the data-points in Z. The new value is denoted by the triangle.

Let $\psi_{j}^{K}$ denote the global ensemble optimized value returned by our model kESVR from among the K candidate prediction values generated by each SVR $S_{k}$. Let $\eta_{k}^{r}\left(e,f\right)$ represents the set of data points in cluster $G_{k}$ that fall within a circle of radius r centered at point (e,f). That is $\eta_{k}^{r}()$ indicates the set of neighbors of point (e,f) within a circle of radius r. So, the neighbors of predicted data point $\left({\bar{Y}}_{jk},\phi_{p}\left(X_{j}\right)\right)$ can be represented as:

(6) $$\eta_{k}^{r}\left({\bar{Y}}_{jk},\phi_{p}\left(X_{j}\right)\right)=\{Z_{l}\}\;s.t.\;Z_{l}\in G_{k}\;and\;Euclidean_{Dist\left(Z_{l},\left({\bar{Y}}_{jk},\phi_{p}\left(X_{j}\right)\right)\right)}\leq r$$

Figure 2f illustrates the concept of $\eta_{k}^{r}()$. The triangle represents the point $\left({\bar{Y}}_{jk},\phi_{p}\left(X_{j}\right)\right)$ and the black circle of radius r is drawn around it. All data points within the circle represent the set $\eta_{k}^{r}\left({\bar{Y}}_{jk},\phi_{p}\left(X_{j}\right)\right)$. In the example shown in Figure 2f, there are 9 points within the radius r, so 9 neighbors.

Let $SP\left(Z_{i},Z_{j}\right)$ denote the Spearman Correlation value between the gene expression values ${\hat{X}}_{i}$ and ${\hat{X}}_{j}$ of the cell-lines $CL_{i}$ and $CL_{j}$ represented by $Z_{i}$ and $Z_{j}$ respectively. We define the β() score of the output ${\bar{Y}}_{jk}$ of SVR $S_{k}$ as follows:

(7) $$\beta \left({\bar{Y}}_{jk}\right)=\frac{\sum SP\left(Z_{l},\left({\bar{Y}}_{jk},\phi_{p}\left(X_{j}\right)\right)\right)}{\left|\eta_{k}^{r}\left({\bar{Y}}_{jk},\phi_{p}\left(X_{j}\right)\right)\right|}\;s.t.Z_{l}\in \;\eta_{k}^{r}\left({\bar{Y}}_{jk},\phi_{p}\left(X_{j}\right)\right)$$

The β() score essentially indicates how similar a data point is to its neighbors based on their gene expression profile. If $\eta_{k}^{r}\left({\bar{Y}}_{jk},\phi_{p}\left(X_{j}\right)\right)=0$ then $\beta \left({\bar{Y}}_{jk}\right)=0$. We select $\psi_{j}^{K}$ as the abscissa value of the prediction data-point, that has the highest $\beta \left({\bar{Y}}_{jk}\right)$ value among all K points. Thus

$$\psi_{j}^{K}={\bar{Y}}_{jk}\;\mathrm{such}\;\mathrm{that}\;\beta \left({\bar{Y}}_{jk}\right)\;\mathrm{is}\;\max.$$

##### Optimal Drug Response Prediction

We return the best value of drug response prediction $\psi_{j}^{K}$ by optimizing the parameters p and K in the following objective function:

(8) $$\;\;\underset{p}{\min}\left(var\left[X\right]-var\left[\phi_{p}\left(X\right)\right]\right)+\underset{K}{\min}\overset{N}{\underset{j=1}{\sum }}\left({\left|Y_{j}-\psi_{j}^{K}\right|}^{2}\right)$$

#### 2.2.3. Steps for Creating kESVR Model for a Specific Drug D

We execute the following steps to develop kESVR model, $kESVR_{D}$ for a particular drug D, using mRNA gene expression data X and drug response data Y.

1. Perform PCA on the mRNA gene expression data X. Use the first principle component (p=1) and create the reduce dataset $Z=\left\{Z_{1},\;Z_{2},\ldots ,\;Z_{N}\right\}$ where $Z_{i}=\left(Y_{i},\phi_{1}\left(X_{i}\right)\right)$.
2. Create the labeled data Q from X and Y. Use 321 target genes ($\left|\hat{X_{i}}\right|=321$) instead of the whole genome data for creating Q.
  $$Q=\left\{Q_{1},\;Q_{2},\ldots ,\;Q_{N}\right\}\;\;\;\mathrm{where}\;Q_{i}=\left\{\left({\hat{X}}_{i},Y_{i}\right)\right\},\;\;\;{\hat{X}}_{i}\subset X_{i}\;\;\mathrm{and}\;\;\left|{\hat{X}}_{i}\right|=321.\;$$
  Train SVR S, on Q (75% training 25% testing data) and record the predicted value errors. From this create the 2-tupled dataset $\Psi =\left\{\left(Y_{1},e_{1}\right),\left(Y_{2},e_{2}\right),\ldots ,\left(Y_{N},e_{N}\right)\right\}$ where $e_{i}$ denotes the prediction error obtained from S for input gene expression ${\hat{X}}_{i}$. Next apply K-means clustering to partition Ψ into K(=12) clusters that can then be used to partition Z into K(=12) clusters $G_{1}\ldots G_{12}$.
3. Repeat for k=1 to 12:
  - Train k SVRs $S_{1}\ldots S_{12}$ on the clusters $G_{1}\ldots G_{12}$ (75% training, 25% testing).
  - Given an input ${\hat{X}}_{j}$, let ${\bar{Y}}_{jk}$ represent the output predicted by $S_{k}$. Calculate the k predicted values from the k SVRs. Then calculate $\beta \left({\bar{Y}}_{jk}\right)$ score for each of the k ${\bar{Y}}_{jk}$.
  - Select the prediction value $\psi_{j}^{K}$ returned by kESVR to input ${\hat{X}}_{j}$ as the value ${\bar{Y}}_{jk}$ with the highest $\beta \left({\bar{Y}}_{jk}\right)$ score.
  - Calculate the Squared Error as ${\left|Y_{j}-\psi_{j}^{K}\right|}^{2}$.
  - Calculate the average Mean Square Error ($MSE_{k}$) for both training and testing data (N cell-lines).
4. Select the value of k with the lowest $MSE_{k}$ among $MSE_{1},..,MSE_{12}$ values as the ideal number of clusters of $kESVR_{D}$.
5. Retain the model created using the optimal value of k (obtained in step 4) as model $kESVR_{D}$ for drug D.

In case of a new gene-expression input (or from X), use the newly created $kESVR_{D}$ to generate predicted drug response value of that input for drug D.

#### 2.2.4. Simulation

**Figure 3:**
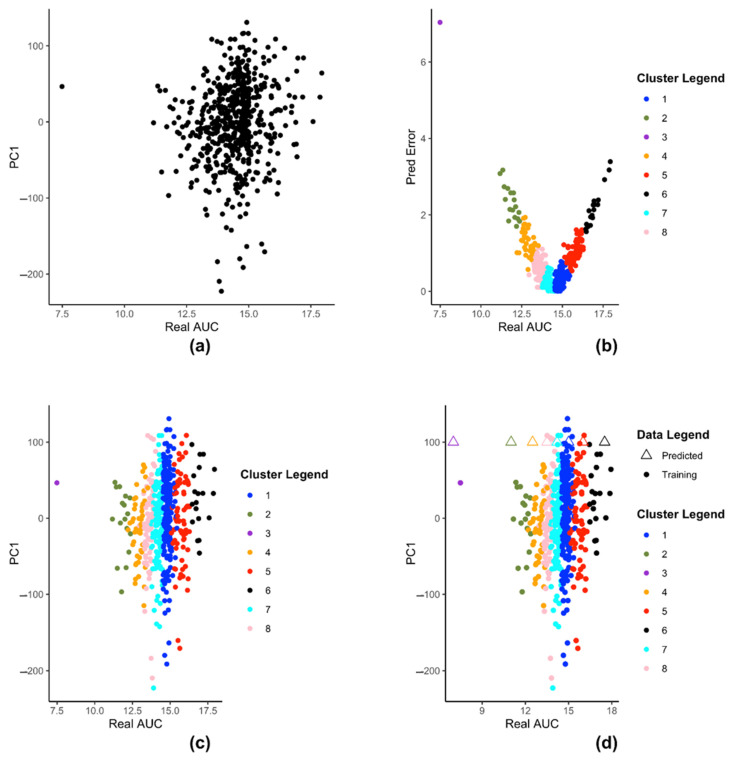
The steps of kESVR model creation for drug zebularine response AUC prediction on 610 cancer cells from CCLE. (**a**) Dimension reduction of gene expression profiles and mapping of the latent variable PC1 and drug response of zebularine onto 2D space. (**b**) Seeking local clusters by k-means algorithm for regression. (**c**) Construction of local SVR regression models after clustering reduced dataset Z into 8 clusters (**d**) Multiple prediction candidates from the trained 8 SVRs on the clusters.

We demonstrate the steps of creation of kESVR model using zebularine drug response from CTRP on 610 cancer cells from CCLE. Figure 3 illustrates the steps for creating the k SVRs in our model development of kESVR for drug zebularine. Figure 3a shows the reduced dataset Z for drug zebularine. Figure 3b plots the data-points in Ψ on a 2-D plane with real/recorded drug AUC values as the abscissa and the errors in the predicted AUC values as the ordinate. Figure 3b is the result of applying K-means clustering on set Ψ that produces 8 clusters. These have been color coded for ease of visualization. Each point in both Figure 3a,b represent the cell lines. We use this clustering information to cluster set Z into the same eight clusters (depicted by the same colors). After clustering of the data points, we train 8 SVRs, one on each cluster. Figure 3c,d shows dataset Z using real drug AUC values as the abscissa and the first principal component of gene expressions as the ordinate. Z is partitioned into 8 clusters (depicted by 8 colors) after which individual SVRs are trained for each cluster. 

Figure 3d shows how kESVR predicts drug response value for a given gene expression input say ${\hat{X}}_{j}$. Each of the eight SVRs, returns a predicted value. These predicted values together with the first principal component value of ${\hat{X}}_{j}$ can be plotted as points on the same 2-D plan containing the clustered data Z. In Figure 3d, these are represented by the triangular points. Note that the color of each triangle matches the color of its generating SVR/cluster. We calculate the β() score for each point. Our kESVR model returns the value (the triangle) with the highest β() score. The idea of the β() score is quite intuitive. While the ordinate value ($\phi_{1}\left({\hat{X}}_{j}\right)$) remains the same for all 8 triangular point, it is the predicted value of each SVR (abscissa value) that determines the location of the triangular point and hence the number of neighbors. To temper the impact of the size of the training set of an SVR (overall density of a cluster), we use the average Spearman correlation value which makes sure that higher similarity of ${\hat{X}}_{j}$ with the genetic profile of a cluster favors the selection of the corresponding predicted value. In other words, if a cluster $G_{i}$ (cell-lines profiles) is more similar to the input ${\hat{X}}_{j}$ than others, then that similarity will be reflected in its β() score.

#### 2.2.5. Implementation

The entire model is implemented using R. We use the caret package [29] to invoke the individual Support Vector Regression models with radial basis function kernel. 

## 3. Results

### 3.1. Comparison with Standard ML Models

We use 610 cancer cell lines from CCLE and 481 drug response of those cell lines from CTRP for testing our kESVR model. We test the performance of kESVR on 5 random drugs (zebularine, azacytidine, myricetin, BRDK64610608 and nelarabine) out of the 481 drugs from CTRP. We compare kESVR with 4 other machine learning models: (i) Linear regression (LR), (ii) Back Propagation Neural network (BPNN), (iii) Support Vector Regression (SVR) and (iv) Quantile Regression Forest (QRF). For each model the labeled dataset Q of 610 cell-lines is split into training and testing (75/25) set. We use the average Mean Square Error (MSE) for both the training and testing dataset are our performance metric. Our kESVR uses 5-fold cross-validation to return the average MSE value for a drug. Table 1, shows the optimal value of k that kESVR uses for each of the drugs. Table 2 compares the average MSE value of each model for the 5 drugs. It is evident that kESVR has the lowest MSE value, making it the best performing model among all the models.

We also compare the model setup time across all the models for each drug. Table 3 shows kESVR takes longer time than LR, SVR or QRF for the model to be setup but its prediction accuracy makes up for the extra time taken in setup. 

We next compare the performance of kESVR against the same 4 models for 5 drugs that have the highest variance in drug AUC values amongst the 481 drugs. These 5 drugs are SB743921, paclitaxel, daporinad, neopeltolide and docetaxel. The idea is to see how well kESVR handles large variance of AUC value for a particular drug across the 610 cell-lines. We use the same metric for evaluation. Table 4 shows that except for drug neopeltolide, our model kESVR performs the best again. Now for 3 drugs, kESVR and SVR seem to have the same MSE value. The reason for this can be found in Table 1.

Table 1 shows the optimal k-value that kESVR uses for each drug. It can be seen that for 3 drugs the value of k is 1. That is, for those drugs after trying out different value of k, kESVR finds k=1 to be the optimal value. A value of k=1 essentially means a single SVR, hence the similarity of MSE values in Table 4. Table 5 like Table 3 shows that kESVR model takes more time than 3 out of the 4 drugs but has higher accuracy than them.

Table 6 and Table 7 gives a glimpse of the performance of our model for drug Zebularine. Table 6 shows that k=8 produces the lowest average MSE value which is why it is selected as the optimal k-value. Table 7 illustrates that our model does not suffer from over-fitting. Detailed results for all 10 drugs can be found in the Supplementary File: Tables S1–S10.

We further compare the goodness of fit of our kESVR model with the 4 models using the R-squared metric. For each drug, we treat the entire data of that drug as our test dataset and use the trained 5 models to generate predicted values for the test data. We then calculate the R-squared value ($R^{2}=1-\left(\mathrm{residual}\;\mathrm{sum}\;\mathrm{of}\;\mathrm{squares}\right)/\left(\mathrm{total}\;\mathrm{sum}\;\mathrm{of}\;\mathrm{squares}\right))$ for each drug and model. It can be seen from Table 8, that for 7 out of the 10 drugs, kESVR has the best fit (>=0.7) among all 5 models. For 3 drugs, the optimal value of k used by kESVR is 1 (Table 6), which is why the R-squared value of kESVR and SVR is the same.

### 3.2. Comparison with Existing Drug Response Prediction Models

**Figure 4:**
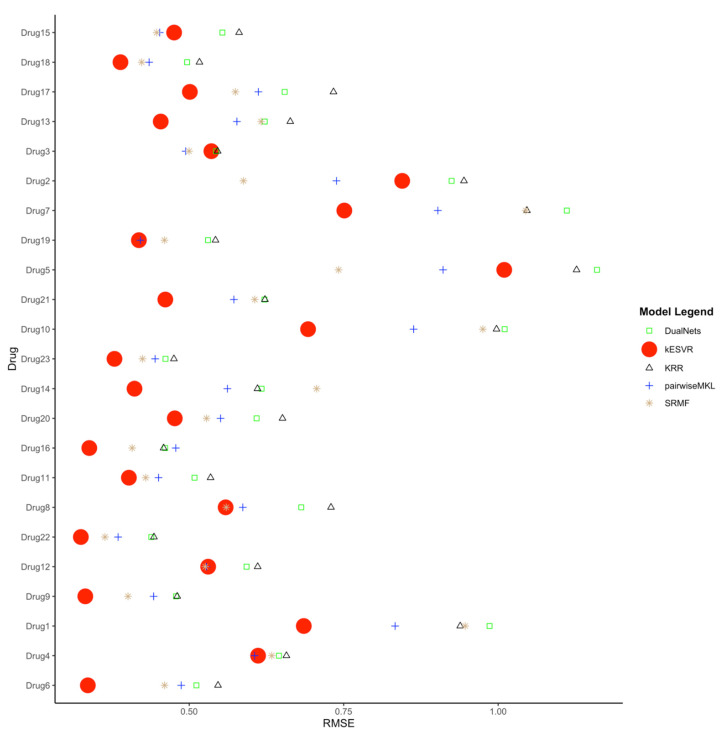
Comparison of kESVR with DualNets, KRR, pairwiseMKL and SRMF models in terms of Root Mean Square Error (RMSE) value over 23 drugs. kESVR is the best performing (lowest RMSE) model in 17 out of 23 drugs. For the remaining 6 drugs, kESVR places in the top 3 position among the 5 models.

We next compare our kESVR model to contemporary drug response prediction computational methods. A paper by Chen and Zhang [11] evaluates and compares the performance of 17 representative methods for drug response prediction developed in the past five years. These include models based on regression and their generalizations, Bayesian inference methods, matrix-factorization methods, random forest, kernel rank learning and deep convolutional neural networks. They assess the performances of these 17 methods using four large public datasets in nine metrics. We use one of the four datasets (CCLE) and one of the metrics (RMSE) used in their paper [11] to compare the methods in terms of prediction accuracy. To ensure fairness of comparison, we use the exact same curated data and the exact same steps that their paper use to generate the Root Mean Square Error (RMSE) values. This curated data contains expression profiles for 385 cancer cell lines and drug response AUC values for 23 drugs. To generate the RMSE value for each drug d, we use five-fold cross-validation; split the data into 5 parts, train the model with 4 parts of data and use the remaining 1 part as test data. After 5-folds, we get the RMSE value for the whole data. We repeat the process for 10 iterations to get the average RMSE value for the drug d. In this manner, we generate the RMSE values for all 23 drugs and compare them with those generated by the other 17 methods. The complete comparison table can be found in the Supplementary File: Table S11. We rank the performance of the 18 methods in terms of RMSE (lower RMSE means better rank) for all 23 drugs in Supplementary File: Table S12. It is evident from the performance rank table that kESVR places in the top 5 positions for all 23 drugs and holds the first rank for 17 out of 23 drugs. That is, for 17 drugs kESVR out-performs all other methods and for the remaining 6 drugs it performs better than at least 12 methods. In their paper, Chen and Zheng conclude that 4 methods: DualNets [30], Kernelized rank recommendation (KRR) [31], pairwise multiple kernel learning (pairwiseMKL) [32] and similarity-regularized matrix factorization (SRMF) [33] are the best among the 17 methods in terms of prediction accuracy. These 4 methods consistently out-perform the other 13 methods. So, we inspect the performance of kESVR against these 4 methods. The full rank table for kESVR and the 4 methods is provided in Supplementary File: Table S13. We plot the RMSE values of kESVR along with those 4 methods in Figure 4. From the plot and the rank table we see that kESVR is the best performing model for 17 out of 23 drugs. In case of the 6 drugs, where it does not rank first, the lowest rank it gets is third and consistently out-performs DualNets and KRR.

## 4. Discussion

kESVR is a data driven model. That is, it does not require any external input of parameter values. kESVR calculates all of its parameters (k, r, parameters of individual SVRs) from the input data directly. This makes this model highly robust.

kESVR uses PCA in its first step. This leads to the creation of the reduced dataset Z (Equation (2)). At this step, there are several principal components to choose from, for our model creation. The decision to use the first principal component for this step stems from the fact that the first principal component retains the maximum percentage of variation in the reduced data set. Graphically this means, it gives a better visualization/separation (Figure 2a) of the distinct data points on the 2-D plot that is used in the subsequent steps.

**Figure 5:**
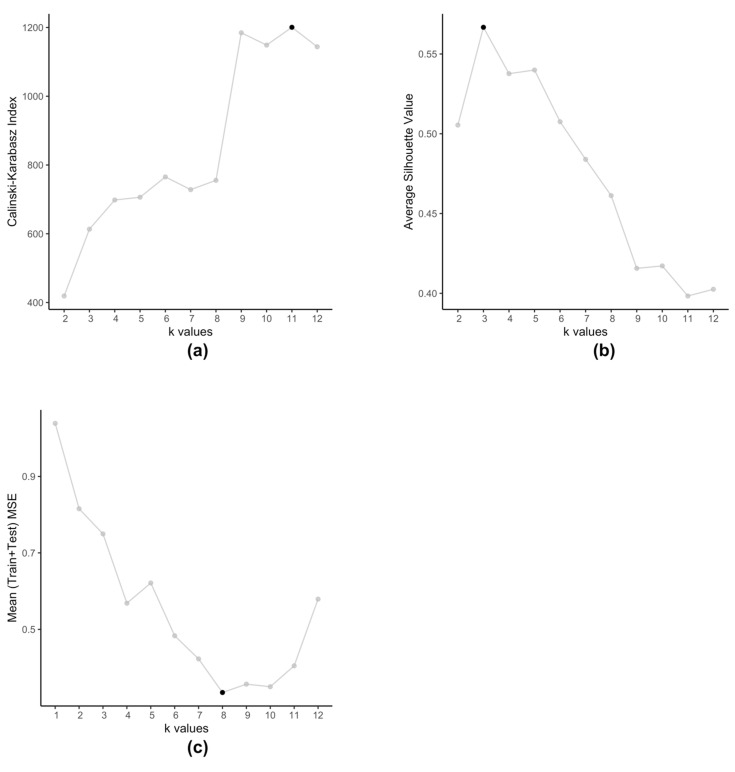
Selection of optimal value of k for drug zebularine. (**a**) Calinski-Karabasz index sets optimal k=11 (**b**) Average Silhouette value specifies optimal k=3 (**c**) Average (Train+Test) MSE value selects optimal k=8.

The embedded clustering step employs K-means clustering on the dataset Ψ (Equation (4)) in order to cluster the cell-lines into groups. We use K-means for two reasons, firstly we plan to cluster cell-lines that produce similar prediction errors and secondly since Ψ is a set of 2-tuple data. These clusters then train separate SVRs to reduce the prediction errors. Typically, K-means method itself is prone to producing different clusters each time due to the randomized nature of its initiation. However, in this particular scenario, we observe that even after running multiple times, with this data K-means always produce the same set of centroids. Traditionally, optimal value of k is determined by using metrics such as Silhouette value [34] or Calinski-Harabasz index [35]. However, such methods are not applicable in case of kESVR as our main objective is not to determine how well the data-points are clustered. Clustering is an intermediate step, that plays an important role in the performance of kESVR. That is, the performance metric MSE is dependent on the choice of k. In that respect the choice of value of k, is data driven. As our final objective is to minimize the value of MSE (Equation (8)), kESVR loops through different values of k, and selects the optimal one that gives the lowest MSE value. Figure 5 illustrates how the optimal value of k varies according to metric used for evaluation. Here we use the model for drug zebularine. Figure 5a uses the Calinski-Harabasz index, Figure 5b uses the average Silhouette value and Figure 5c uses the average (Training+Testing) MSE values to get optimal k for drug zebularine. Accordingly, the suggested optimal value of k turn out to be 11, 3 and 8 respectively. From Table 6, we known that only k=8 gives the lowest average MSE value (best kESVR performance). So k=8 is selected as the optimal value.

**Figure 6:**
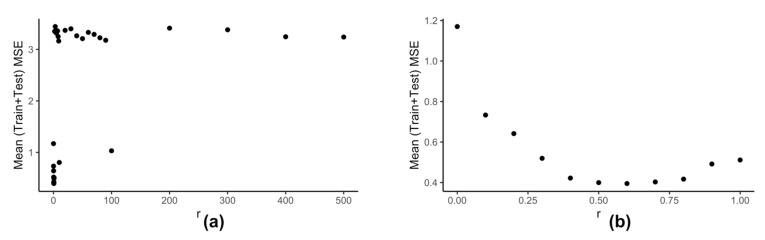
Variation of performance of kESVR with respect to r for drug zebularine. (**a**) *r* is varied from 0 to 500. (**b**) Shows the details from (a) when r is varied from 0 to 1.

Computation of neighbors for any data-point using $\eta_{k}^{r}()$ (Equation (6)) is dependent on the value of r. We use the clustering information of the reduced data Z to calculate the best value of r. Our model kESVR is data-driven. Being an ensemble method, its performance is dependent on the size/volume of the training data being used on each individual SVR. That is, some clusters in Z can be denser than others. Depending on the value of r, that density of a cluster can influence the overall performance of kESVR. Figure 6 shows how the performance of kESVR varies with r for drug zebularine. 

We can see from Figure 6a that as the value of r increases the performance of our model deteriorates. This observation is intuitive: if a cluster is very dense (more training instances) and the value of r is sufficiently large, then that cluster can end up dominating the prediction value selection process and hence the overall performance. The best performance is given when r is small. This is shown is Figure 6b. Keeping these things in mind we calculate r as follows: We calculate the x-axis distance (AUC values) between the two farthest points for each cluster. We sort these distances in ascending order and select r to be smallest value. This is done in order to be fair in comparing the number of neighbors for each cluster. If in a particular prediction instance, a value of r results in zero neighbors in all clusters, we simply select the next higher value in the sorted list, and redo the neighbor selection process for that instance. 

kESVR uses an ensemble approach to return the final prediction value from k potential predicted values. It uses a maximum β() score (Equation (7)) approach wherein the potential predicted value with the highest β() score is selected as a final prediction value. We empirically test a total of five different approaches before settling on the β() score one. The four other approaches are:

(i) select the prediction value whose data-point has the maximum average Spearman correlation value with all cell-lines in the training set of its parent SVR. 

(ii) return a weighted average of the k prediction values using the β() scores of the corresponding data-points as weights.

(iii) select the prediction value whose data-point has the maximum number of neighbors (using $\eta_{k}^{r}()$).

(iv) return a weighted average of the k prediction data-points using the number of neighbors (from $\eta_{k}^{r}()$) of the corresponding data-points as weights.

Our experimental comparisons show that the maximum β() score approach gives the best accuracy performance i.e. lowest MSE values. 

Lastly feature selection plays an important role in the performance of our model kESVR. Intuitively, features indicate those genes that play a crucial role in the functioning of a drug. Expression (high/low) of these feature genes, affects how a cell-line/patient responds to the drug. We use genes that are known to be target genes for the drugs (available from public databases) as our feature genes. This contributes to the improved performance of kESVR over other drug response prediction methods.

## 5. Conclusions

In this paper we introduce a novel computational model for drug sensitivity prediction. We show that our model kESVR consistently outperforms existing traditional ML tools and at least 12 recently developed drug response prediction methods in terms of prediction accuracy. Even among the 4 good methods deemed best performing by Chen and Zhang [11] it is the best performing model in most cases. It is a robust model that is completely data driven and combines both supervised and unsupervised learning concepts in its functionality. 

As of now kESVR only uses gene expression data. However, as future work we plan to extend our kESVR model, to incorporate other kinds of omics data such as mutation, copy number variation and methylation data. Currently kESVR does not allow for feature selection. This is something that we will be working on, so that future users can use kESVR to get bio-markers for the drugs as well as their response prediction results. Finally, we plan to use kESVR to create a precision medicine drug recommendation system for patients wherein we use cancer lines and other omics data to perform drug response prediction and drug ranking recommendation efficiently.

## Figures and Tables

**Table 1:** Optimal k-value for kESVR model.

Drug	Optimal k
zebularine	8
azacitidine	7
myricetin	8
BRDK64610608	8
nelarabine	12
SB743921	1
paclitaxel	8
daporinad	8
neopeltolide	1
docetaxel	1

**Table 2:** Model comparison for 5 random drugs.

Drug	Avg. (Training + Testing) MSE				
	LR	BPNN	SVR	QRF	kESVR
zebularine	36.490	1.078	1.039	1.001	0.336
azacitidine	188.773	0.983	1.028	1.001	0.307
myricetin	117.890	0.902	0.905	0.984	0.301
BRDK64610608	49.670	0.987	1.018	1.078	0.350
nelarabine	42.137	1.010	1.093	1.090	0.450

**Table 3:** Model setup time for 5 random drugs.

Drug	Model Setup Time (in sec)				
	LR	BPNN	SVR	QRF	kESVR
zebularine	2.390	9417.563	30.328	3342.786	10,934.530
azacitidine	2.419	8830.332	29.412	3603.740	7787.4456
myricetin	2.487	15,179.391	30.088	3375.857	8259.927
BRDK64610608	2.493	13,580.990	29.556	3329.557	8442.622
nelarabine	2.335	14,683.006	27.803	3608.870	7334.934

**Table 4:** Model comparison for 5 drugs with maximum variance.

Drug	Avg. (Training + Testing) MSE				
	LR	NN	SVR	QRF	kESVR
SB743921	674.143	3.496	3.095	3.238	3.095
paclitaxel	98.3038	3.442	3.067	3.070	2.472
daporinad	137.176	3.458	3.206	3.140	2.082
neopeltolide	118.476	3.256	3.358	3.443	3.358
docetaxel	110.085	3.360	2.856	3.074	2.856

**Table 5:** Model setup time for 5 drugs with maximum variance.

Drug	Model Setup Time (in sec)				
	LR	BPNN	SVR	QRF	kESVR
SB743921	2.271	10,361.546	27.447	3563.221	8918.989
paclitaxel	2.088	9439.555	27.139	3497.768	8575.110
daporinad	2.316	11,495.089	26.572	2391.248	7637.791
neopeltolide	1.617	2132.497	7.937	570.632	2738.013
docetaxel	1.650	2849.202	11.419	1139.292	4266.580

**Table 6:** Selection of optimal k value for kESVR model development of drug zebularine.

DRUG Zebularine	
*k*	Avg. (Training + Testing) MSE
1	1.039
2	0.815
3	0.749
4	0.568
5	0.621
6	0.483
7	0.423
8	0.336
9	0.357
10	0.351
11	0.405
12	0.579

**Table 7:** 5-fold cross-validation results for kESVR model (*k* = 8) of drug zebularine.

DRUG Zebularine			
Fold	Training Set MSE	Testing Set MSE	Avg. (Training + Testing) MSE
1	0.203	0.335	0.269
2	0.063	0.706	0.384
3	0.207	0.618	0.413
4	0.227	0.276	0.252
5	0.316	0.404	0.360

**Table 8:** Model Fitness Comparison using R-squared values.

Drug	*R*—Squared Value				
	LR	BPNN	SVR	QRF	kESVR
zebularine	−0.094	−0.00047	0.284	0.693	0.778
azacitidine	−0.267	−4.5 × 10^−5^	0.139	0.691	0.789
myricetin	−0.25	−0.000681	0.082	0.698	0.772
BRDK64610608	0.072	−0.000809	0.093	0.698	0.802
nelarabine	−0.152	−4.2 × 10^−5^	0.119	0.627	0.700
SB743921	0.263	−0.002453	0.66	0.795	0.66
paclitaxel	0.093	−6.0 × 10^−6^	0.415	0.764	0.853
daporinad	−0.262	−0.000608	0.382	0.768	0.903
neopeltolide	−11789.886	−0.00368	0.385	0.781	0.385
docetaxel	−301.018	−0.001727	0.644	0.788	0.644

## Data Availability

Functional genomics data in this manuscript can be found in: Gene expression of breast cancer cell lines are from Cancer Cell Line Encyclopedia (CCLE) (http://www.broadinstitute.org/ccle); Large scale of drug screening in cancer cells is from the Cancer Therapeutics Response Portal (CTRP, https://portals.broadinstitute.org/ctrp). Curated data used in method comparison can be found in the GitHub link provided by the paper by Chen and Zhang [11]. Users can access kESVR model for free at https://github.com/abhishekmaj08/kESVR.git.

## Supplementary Materials

The following are available online at https://www.mdpi.com/article/10.3390/genes12060844/s1. Supplementary Table S1: 5-fold cross-validation results of kESVR on zebularine for *k* = 2:12, Supplementary Table S2: 5-fold cross-validation results of kESVR on azacitidine for *k* = 2:12, Supplementary Table S3: 5-fold cross-validation results of kESVR on myricetin for *k* = 2:12, Supplementary Table S4: 5-fold cross-validation results of kESVR on BRDK64610608 for *k* = 2:12, Supplementary Table S5: 5-fold cross-validation results of kESVR on nelarabine for *k* = 2:12, Supplementary Table S6: 5-fold cross-validation results of kESVR on SB743921 for *k* = 2:12, Supplementary Table S7: 5-fold cross-validation results of kESVR on paclitaxel for *k* = 2:12, Supplementary Table S8: 5-fold cross-validation results of kESVR on daporinad for *k* = 2:12, Supplementary Table S9: 5-fold cross-validation results of kESVR on neopeltolide for *k* = 2:12, Supplementary Table S10: 5-fold cross-validation results of kESVR on docetaxel for *k* = 2:12, Supplementary Table S11: Comparison of RMSE values of kESVR with 17 other methods across 23 drugs on curated CCLE dataset, Supplementary Table S12: Performance rank of kESVR with 17 representational methods across 23 drugs on curated CCLE dataset, Supplementary Table S13: Performance rank of kESVR, DualNets, KRR, pairwiseMKL and SRMF across 23 drugs on curated CCLE dataset

## Abbreviations

Area under the curve (pharmacokinetics)(AUC); Back Propagation Neural network (BPNN); Cancer Cell Line Encyclopedia (CCLE); Cancer Therapeutics Response Portal (CTRP); k-means Ensemble Support Vector Regression (kESVR); Kernelized Rank Recommendation (KRR); Next Generation Sequencing (NGS); Reverse-Phase Protein Array (RPPA); linear regression (LR); Quantile Regression Forest (QRF); Root Mean Square Error (RMSE); Support Vector Regression (SVR); Similarity-Regularized Matrix Factorization (SRMF); Whole-Genome Sequencing (WGS).
